# Intrauterine midgut volvulus as a rare cause of intestinal obstruction: a case report

**DOI:** 10.1186/s13256-021-02778-6

**Published:** 2021-05-04

**Authors:** Gonca Gerçel, Ali İhsan Anadolulu

**Affiliations:** 1 Department of Pediatric Surgery, Şanlıurfa Training and Research Hospital, Şanlıurfa, Turkey; 2 Department of Pediatric, Şanlıurfa Mehmet Akif İnan Training and Research Hospital, Şanlıurfa, Turkey

**Keywords:** Case report, Intrauterine midgut volvulus, Rare

## Abstract

**Background:**

Intrauterine midgut volvulus is a very rare, life-threatening condition, and prenatal diagnosis is difficult. In this article, we present a case of midgut volvulus followed by a pre-diagnosis of antenatal jejunal atresia.

**Case presentation:**

A 1-day-old Turkish male baby, who was followed with a diagnosis of antenatal jejunal atresia, with a birth weight of 3600 g, delivered by cesarean section at 38 weeks of gestation from a 19-year-old mother in her fourth pregnancy, was taken to the newborn intensive care unit. The patient underwent surgery on the postnatal first day with a preliminary diagnosis of jejunal atresia. It was observed that the small intestine was rotated two full cycles from the mesenteric root. Bowel blood circulation was good. Volvulus was untwisted. There was malrotation. Ladd's procedure was performed. The baby was discharged on the seventh postoperative day with full oral feeding. The patient is still in the first postoperative year and follow-up has been uneventful.

**Conclusion:**

Intrauterine midgut volvulus has been associated with high mortality in the literature. Differential diagnosis of midgut volvulus in patients with antenatal intestinal obstruction, close prenatal follow-up, appropriate delivery and timing of surgical intervention may significantly reduce morbidity and mortality.

## Background

Small bowel volvulus occurs when bowel loops become twisted around the mesenteric artery or its branches. Midgut volvulus is common in infancy but is an extremely rare life-threatening condition during fetal development [1–3]. The twisting of the mesenteric artery leads to vascular congestion, impaired venous return and bowel necrosis [4]. It is a surgical emergency, and delay in diagnosis or treatment can increase fetal morbidity and mortality. Prenatal diagnosis of this type of malformation can be difficult in the absence of specific signs [5].

In this article, we present a case of midgut volvulus that was followed up with a pre-diagnosis of antenatal jejunal atresia starting from the antenatal period. This report is aimed at increasing awareness regarding patients with a diagnosis of fetal bowel dilatation, because it is a very rare condition, and timely intervention could be life-saving.

## Case presentation

A 19-year-old Turkish mother in her fourth pregnancy was admitted to our institute with an ultrasound report suggestive of dilated small bowel loops at 24 weeks of gestation, which was followed up in the department of maternal fetal medicine with a pre-diagnosis of fetal bowel obstruction. The mother’ s previous obstetrical history was uneventful. There was no relationship between the parents. Repeat ultrasonography revealed a segmental distended bowel, with preserved motility and vascularization. There was no evidence of fetal anemia, and the amniotic fluid index was within the normal range. Jejunal atresia was considered as the first differential diagnosis in the fetus.

**Fig. 1:**
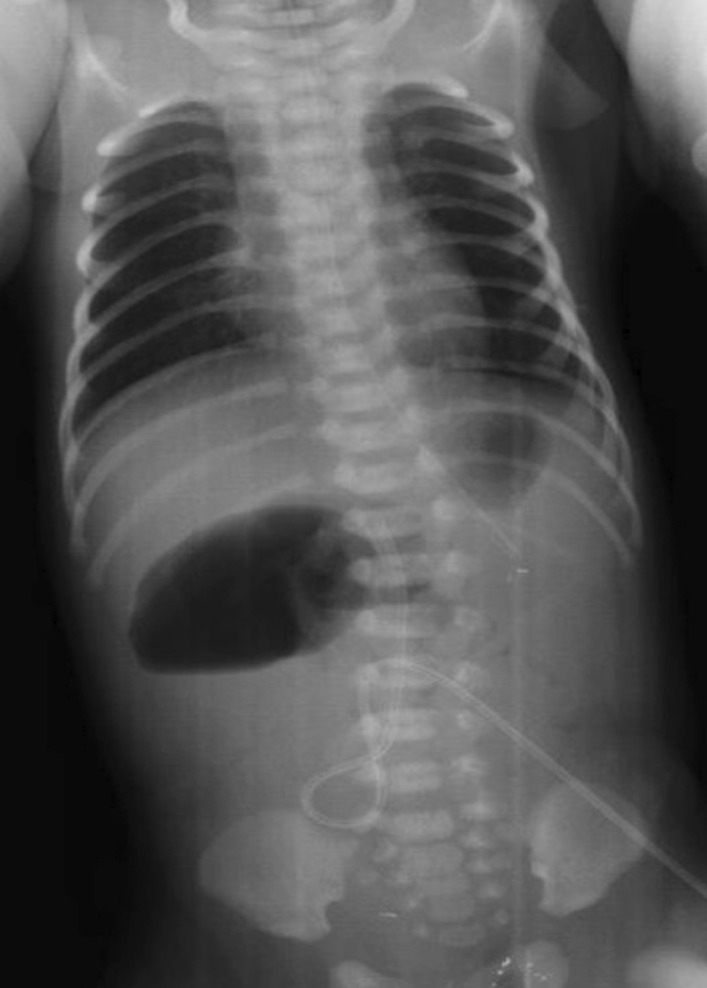
Air–fluid levels suggesting proximal obstruction on X-ray

At 38 weeks of gestation, the mother underwent a planned cesarean section due to a history of previous cesarian section. A Turkish male child weighing 3600 g was delivered, with APGAR scores of 8 and 10 at 1 and 5 minutes, and pulse, blood pressure and temperature within normal ranges. The baby was immediately transferred to the intensive care unit under pediatric surgery. An infant feeding tube was secured in place. Bilious drainage from a nasogastric catheter was detected. On physical examination, the patient was alive and active. The abdomen was soft and had minimal distention. Neurological examination was normal. Septic findings were not observed in the patient. Laboratory values of the patient were within the normal range. An initial complete blood count revealed no anemia or leukocytosis, with hemoglobin of 15.2 g/dL, platelet clumping with a normal count (221,000/uL) and white blood count of 16,200/uL. There was no meconium output in the follow-up. In radiological evaluation, there was no distal gas passage on the abdominal X-ray, and air–fluid levels suggested proximal obstruction (Fig. 1).

**Fig. 2:**
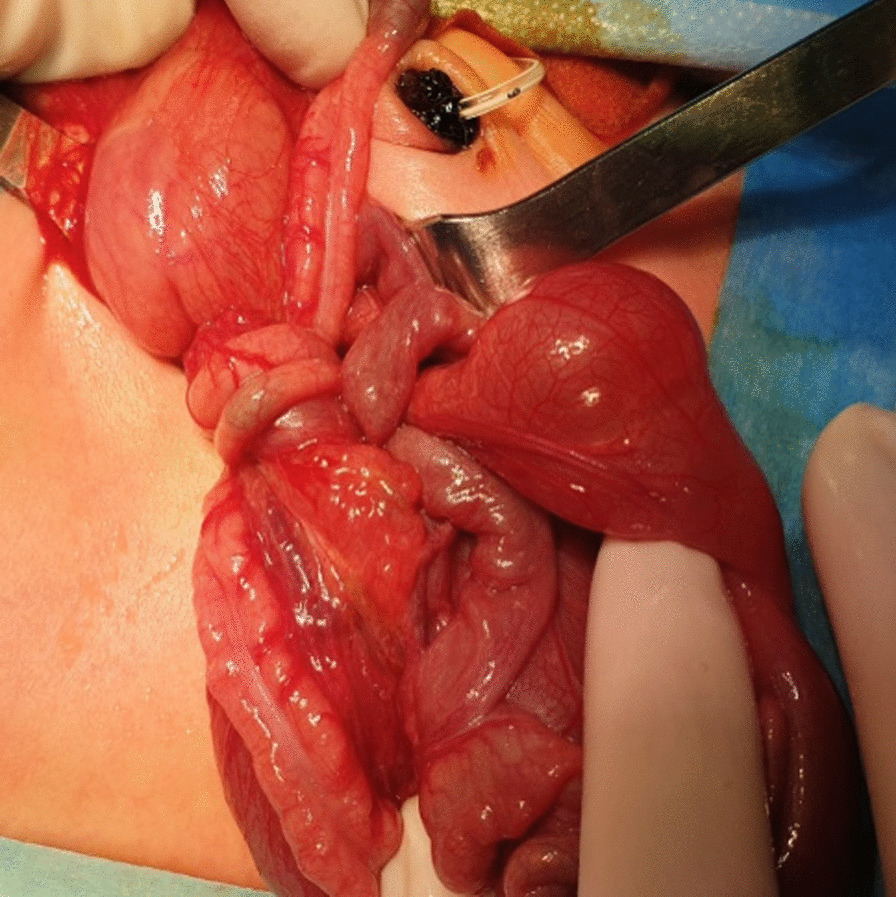
The view of the intestines rotated two full cycles (720°) from the mesenteric root

**Fig. 3:**
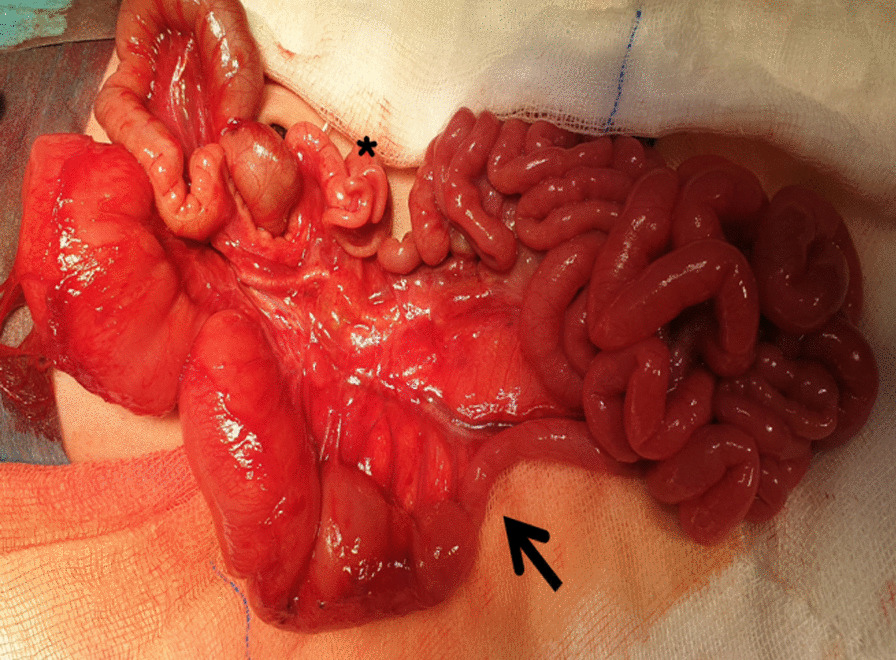
Small bowel segment diameter difference (arrow), appendix vermiformis (asterisk)

The patient underwent surgery on the first postnatal day. In laparotomy, it was observed that the small intestine was rotated two full cycles from the mesenteric root (Fig. 2). Bowel blood circulation was good. Volvulus was untwisted. There was a significant difference in diameter in the small bowel segment approximately 20 cm distal to Treitz. No atresia was detected. It was thought to be due to Ladd’s bands compression (Fig. 3). There was malrotation. A Ladd's procedure was performed. The patient was started on oral feeding on the first postoperative day. There was no problem in follow-up. He was discharged on the seventh postoperative day with full oral feeding. The patient is still in the first postoperative year, and the follow-up has been uneventful.

## Discussion

The incidence of malrotation has been estimated at 1 in 6000 live births [6]. The most serious consequence of malrotation is volvulus [7]. Midgut volvulus is a rare condition. It occurs when a bowel loop twists around its mesenteric pedicle, thereby causing intermittent closed-loop bowel obstruction with or without bowel ischemia [7].

Prenatally diagnosed midgut volvulus remains a rare entity, seldom described in the literature [8] 9. Although volvulus with malrotation usually occurs in the late neonatal period, most cases of *in utero* volvulus occur without malrotation [8]. However, as in the case we presented, there may be cases of midgut volvulus accompanied by malrotation antenatally.

With early recognition and diagnosis of intestinal volvulus, a plan for neonatal surgery before fetal deterioration is extremely important, as there are reports in the literature of fetal demise *in utero* or postnatal death due to late intervention [4]. However, it is still challenging because of a lack of specific fetal symptoms or ultrasonographic signs.

There are various ultrasonographic signs of fetal intestinal volvulus, including dilated aperistaltic bowel loops, whirlpool sign or coffee bean sign [9]. Other indirect findings that may present in intestinal volvulus are ascites, discrete cystic or solid abdominal mass, peritoneal calcification, polyhydramnios, and particularly decreased fetal motion or abnormal fetal cardiotocography findings [4, 10].

Although the abovementioned ultrasonographic findings for volvulus have been described, most of the patients are followed up as a diagnosis of fetal bowel dilatation without making a specific diagnosis. A retrospective study by Huang *et al*. described 68 cases with prenatal diagnosis of fetal bowel dilatation. They found that the congenital gastrointestinal tract anomalies included 24 cases (92.3%) of intestinal atresia, one case (3.85%) of small intestine volvulus and one case (3.85%) of malrotation [11]. In the case we presented, there was only fetal bowel dilatation. Bowel motility and vascularization were normal on Doppler studies. No signs of rotation were detected. Therefore, intestinal atresia was considered as a preliminary diagnosis.

It is extremely difficult to predict the time of volvulus and prognosis of the affected fetus owing to a wide range of presentation, from fetal demise to survivor with good outcome. Fortunately, although the case we presented healed with a good result, there are many cases in the literature of fetal demise due to intrauterine volvulus [12, 13]. Currently, the American College of Obstetricians and Gynecologists and the American College of Radiology recommend routine sonograms in the first and/or second trimester, but state that no evidence exists to recommend routine sonograms in the third trimester [14]. Bawa and Kannan demonstrated different outcomes of fetal intestinal volvulus based on the performance of prenatal scanning, and suggested that a single third trimester scan for fetal anomalies may be an effective strategy to reduce perinatal mortality [15]. We believe that patients with fetal bowel dilatation should be closely followed in the third trimester.

## Conclusion

Prenatal suspicion and/or diagnosis of intestinal volvulus is essential for newborn outcome, given the association with high mortality in the literature. Although the presented case had good results, differential diagnosis of midgut volvulus in patients with antenatal intestinal obstruction, close prenatal follow-up, and appropriate delivery and timing of surgical intervention may significantly reduce morbidity and mortality.

## Data Availability

All patient data are accessible from the Republic of Turkey Ministry of Health system.
